# Parents’ Perceptions of Their Parenting Journeys and a Mobile App Intervention (Parentbot—A Digital Healthcare Assistant): Qualitative Process Evaluation

**DOI:** 10.2196/56894

**Published:** 2024-06-21

**Authors:** Joelle Yan Xin Chua, Mahesh Choolani, Cornelia Yin Ing Chee, Huso Yi, Yiong Huak Chan, Joan Gabrielle Lalor, Yap Seng Chong, Shefaly Shorey

**Affiliations:** 1 Alice Lee Centre for Nursing Studies Yong Loo Lin School of Medicine National University of Singapore Singapore Singapore; 2 Department of Obstetrics and Gynaecology National University Hospital Singapore Singapore; 3 Department of Psychological Medicine National University Hospital Singapore Singapore; 4 Saw Swee Hock School of Public Health National University of Singapore Singapore Singapore; 5 Biostatistics Unit Yong Loo Lin School of Medicine National University of Singapore Singapore Singapore; 6 School of Nursing and Midwifery Trinity College Dublin Dublin Ireland

**Keywords:** perinatal, parents, mobile app, chatbot, qualitative study, interviews, experiences, mobile phone

## Abstract

**Background:**

Parents experience many challenges during the perinatal period. Mobile app–based interventions and chatbots show promise in delivering health care support for parents during the perinatal period.

**Objective:**

This descriptive qualitative process evaluation study aims to explore the perinatal experiences of parents in Singapore, as well as examine the user experiences of the mobile app–based intervention with an in-built chatbot titled Parentbot—a Digital Healthcare Assistant (PDA).

**Methods:**

A total of 20 heterosexual English-speaking parents were recruited via purposive sampling from a single tertiary hospital in Singapore. The parents (control group: 10/20, 50%; intervention group: 10/20, 50%) were also part of an ongoing randomized trial between November 2022 and August 2023 that aimed to evaluate the effectiveness of the PDA in improving parenting outcomes. Semistructured one-to-one interviews were conducted via Zoom from February to June 2023. All interviews were conducted in English, audio recorded, and transcribed verbatim. Data analysis was guided by the thematic analysis framework. The COREQ (Consolidated Criteria for Reporting Qualitative Research) checklist was used to guide the reporting of data.

**Results:**

Three themes with 10 subthemes describing parents’ perceptions of their parenting journeys and their experiences with the PDA were identified. The main themes were (1) new babies, new troubles, and new wonders; (2) support system for the parents; and (3) reshaping perinatal support for future parents.

**Conclusions:**

Overall, the PDA provided parents with informational, socioemotional, and psychological support and could be used to supplement the perinatal care provided for future parents. To optimize users’ experience with the PDA, the intervention could be equipped with a more sophisticated chatbot, equipped with more gamification features, and programmed to deliver personalized care to parents. Researchers and health care providers could also strive to promote more peer-to-peer interactions among users. The provision of continuous, holistic, and family-centered care by health care professionals could also be emphasized. Moreover, policy changes regarding maternity and paternity leaves, availability of infant care centers, and flexible work arrangements could be further explored to promote healthy work-family balance for parents.

## Introduction

### Background

Many parents feel stressed during the perinatal period (duration from conception to 1 year postpartum), putting them at risk for developing psychological problems such as perinatal anxiety and depression [1,2]. Studies have reported that approximately between 26% and 15% of mothers [3,4] and 8% to 14% and 11% of fathers [5,6] experience perinatal depression and anxiety, respectively. Moreover, a previous study conducted in Taiwan reported an increasing trend of stress, anxiety, and depression levels among women from around 24 gestational weeks to 1 month post partum, indicating that parents would benefit from more support during this crucial transition to the parenting period [7]. Moreover, the signs of postpartum depression and anxiety tend to be evident within 1 to 3 months after childbirth [4,8-10]. Poor mental health among parents can cause adverse effects on their child’s physical, social, emotional, cognitive, and psychological development [11-14]. These effects are not only evident during infancy but also in their childhood and adolescent years [11,15,16]. In Singapore, approximately 40% of mothers have reported experiencing perinatal depressive symptoms [17], and fathers have also expressed feelings of distress [18]. In addition, parents in Singapore have found it especially difficult and stressful to adjust to life with their new babies within the first month post partum [19]. Moreover, a recent study reported that Singaporean fathers are negatively affected by their partners’ poor mental health during the first 3 months post partum [20]. Given that parents in Singapore are at risk for mental health problems, there is an increased need to understand parents’ experiences with perinatal care and provide them with needed support.

As mobile health apps can provide health care support swiftly and opportunely to many individuals concurrently [21-23], the World Health Organization has promoted their development and use to encourage self-care [24]. Since the functions of mobile apps can be tailored to meet users’ specific needs, researchers have developed mobile apps to help individuals with various medical conditions such as cancer, depression, anxiety, and hypertension [25-27]. A recent review also found that specially developed perinatal mobile apps were promising in providing informational, social, and mental health support to parents during the perinatal period [28]. Considering that most of Singapore’s population are smartphone users [29], conducting a mobile app–based intervention for parents in Singapore is viable.

Previous mobile app–based interventions provided parents with multimedia educational resources and discussion forums to connect with peers and health care professionals [30-32]. Parents appreciated the convenient and trustworthy information about perinatal care provided by these mobile app–based interventions [19,33,34]. In addition, they valued receiving helpful parenting tips from peers and having their queries answered by local health care professionals [19,33,34]. However, parents wanted faster answers to their queries [33,34], and some suggested using chatbots in future mobile apps [19].

Chatbots are digital programs that communicate with humans using voice, text, or animation and can provide instant support to individuals in real time [35]. They have been reported to be capable of providing parents with valuable educational, socioemotional, and mental health support during the perinatal period [36]. The anonymous and nonjudgmental nature of chatbots could also help to connect with individuals who may be hesitant to interact with their peers or health care professionals, even via a digital anonymous platform [37]. Therefore, a mobile app–based intervention with an in-built chatbot titled Parentbot–a Digital Healthcare Assistant (PDA) was developed and implemented to provide more efficient perinatal support to parents [38]. The PDA is a multicomponent mobile app–based intervention that provides parents with informational, psychological, and social support during pregnancy (>24 weeks of gestation) till the first month post partum. The quantitative evaluation of the PDA’s effectiveness on parenting outcomes including parenting self-efficacy, stress, anxiety, depression, and social support has been conducted and reported under the trial outcomes (article under review), and this study aimed to present the in-depth exploration of parental views about the intervention via process evaluation. A process evaluation would allow the thorough examination of the various aspects of intervention delivery and receipt as well as the mechanisms through which the intervention can bring about change and identify the barriers and facilitators that affected intervention delivery and experience [39]. A qualitative approach was selected to better collect descriptive insights about the parents’ experiences and allow subsequent intervention methods to be tailored to the parents’ specific needs [40,41].

### Aims

This study aimed to explore the perinatal experiences of parents and examine the user experiences of the PDA over the same period (from >24 gestational weeks, age of viability in Singapore until 1 month post partum). The perinatal experiences of both the control and intervention groups were included to compare and differentiate their experiences to gain holistic insights related to the PDA. The research questions are as follows: (1) What are the perceptions of parents who received standard perinatal care in Singapore? (2) What are the experiences of parents who received the PDA in addition to standard perinatal care in Singapore?

## Methods

### Research Design

A descriptive qualitative study design was used to analyze and interpret the data that closely captured the participants’ raw experiences [42]. This study’s findings were reported according to the COREQ (Consolidated Criteria for Reporting Qualitative Research) checklist [43]. This study took place in a tertiary hospital in Singapore from November 2022 to August 2023. The study was part of a randomized controlled trial (RCT) that investigated the effectiveness of the PDA on 7 perinatal parenting outcomes: parenting self-efficacy, stress, anxiety, depression, social support, parent-child bonding, and parenting satisfaction (article under review). The PDA is a multicomponent mobile app–based intervention that provides both first-time and experienced parents (mothers and fathers) with educational and psychosocial support from >24 gestational weeks (age of viability in Singapore) until 1 month post partum. It includes multimedia educational resources, a rule-based chatbot, a discussion forum, expert advice, and gamification features such as digital badges. Most of the content in the PDA is relevant for both first-time and experienced parents. However, some resources specifically catered to experienced parents (eg, managing sibling rivalry and engaging older children to care for the newborn sibling). More details of the PDA can be found in the published development paper by Chua et al [38].

### Participants

A total of 118 heterosexual couples were recruited from a tertiary public hospital offering maternity care to the central region of Singapore in the main RCT. The hospital has >1200 beds with >50 clinical specialties. After obtaining written informed consent, couples were randomly assigned to the intervention or control group. Couples in the control group received standard perinatal care provided by the hospital in the form of hospital visits and appointments with their respective obstetricians. By contrast, couples in the intervention group received access to the PDA from the point of study recruitment (>24 weeks of gestation) until 1 month post partum, in addition to the standard perinatal care provided by the hospital. Data for the main RCT were collected at baseline (at the time of recruitment and consent taking) and at the first- and third-month postpartum period. Immediately after the PDA intervention at 1 month post partum, fathers or mothers from different couples were invited from both the intervention and control groups to explore their experiences with the PDA and standard perinatal care received. Parents were interviewed one-on-one without their partners to elicit more honest responses from them [44], and fathers and mothers from different couples were interviewed to obtain more variation in their perinatal experiences.

The eligibility criteria for parents to be included in the qualitative study were if they (1) were aged ≥21 years, (2) were able to read and speak English, (3) gave birth to a newborn without serious medical complications (eg, congenital anomalies or genetic disorders), (4) stayed in Singapore for the 3 months after childbirth, and had varied mean parenting self-efficacy scores as measured by the 10-item Parenting Efficacy Scale (PES) at baseline (>24 gestational weeks) [45]. Items of the PES were rated on a 4-point Likert scale ranging from 1 to 4, with higher total scores indicating a higher level of perceived parenting self-efficacy [45]. An example of an item is “How good do you feel you are at feeding your baby?” [45]. A high internal consistency with a Cronbach α of 0.91 was reported for the PES at the baseline time point among all parents recruited in the main RCT. A purposive sample of 20 parents with varied PES scores (mothers: n=10, 50%; fathers: n=10, 50%) participated in this study. Data saturation was achieved at the 18th participant when no new findings emerged [46], and 2 additional interviews were conducted to confirm the findings.

### Data Collection

Parents’ sociodemographic characteristics and childbirth details were collected using web-based surveys. A semistructured interview guide was developed based on literature [19,33] and the multidisciplinary study team’s expertise (eg, nurses, obstetricians, and midwives). To ensure the comparability of data, parents from the intervention and control groups were asked the same questions regarding their experience with standard perinatal care. In addition, parents from the intervention group were asked about their personal experience with using the PDA, while parents from the control group were asked about their views regarding telehealth services for perinatal care. Open-ended questions, which allowed parents to freely share their views with little restrictions (eg, What were the challenges faced during the perinatal period? What were your experiences with using the PDA?), guided the main structure of the interview [47,48], while follow-up and probing questions were asked to allow researchers to elicit clarity and further insights from parents [49]. The one-on-one interviews were conducted via the web-based Zoom platform (Zoom Video Communications; as per participants’ preferences) at the end of the intervention (ie, after 1 month post partum) and were audio recorded. All interviews were conducted by the primary researcher (JYXC), a female, year-2 PhD nursing student who had been trained in qualitative interviewing techniques by an experienced qualitative nurse researcher and midwife (SS). Field notes of the parents’ nonverbal cues were also documented after each interview. The interviewer (JYXC), with a maternity care background, was trained by the experienced qualitative researcher (SS) to document information regarding parents’ tone of voice, facial expressions, movements, hand gestures, and emotional expressions during the interview. Data collection lasted over a period of 5 months from February to June 2023. The average duration of interviews was approximately 53 (SD 14) minutes for the control group and 49 (SD 12) minutes for the intervention group. All the recordings were transcribed verbatim by research assistants and checked for accuracy by the primary researcher.

### Data Analysis

Descriptive statistics (mean and SD for continuous variables and frequency and percentage for categorical outcomes) were used to summarize parents’ sociodemographic characteristics and childbirth details. The 6-step qualitative thematic analysis framework proposed by Braun and Clarke [50] guided the data analysis process: (1) familiarizing oneself with the collected data, (2) generating initial codes, (3) searching for themes, (4) reviewing themes, (5) defining and naming themes, and (6) producing the report [50]. All transcripts and field notes were read numerous times to gain familiarity with the data set. Next, a manual color-coding method was used to generate the initial set of codes before organizing them into common themes. Codes from the intervention and control groups were compared and tallied to triangulate the data. The initial coding process on all the collected data was conducted by the primary researcher (JYXC) independently, and frequent meetings were held with the research team to discuss the emerging themes. During team meetings, discrepancies in researchers’ judgments were resolved, and the themes were confirmed to ensure that the analysis process was credible.

### Rigor

Rigor was maintained throughout the study by ensuring credibility, dependability, transferability, and confirmability [51,52]. To ensure credibility, transcripts were read and reread numerous times to confirm that the derived themes represented parents’ experiences [51,52]. The provision of thick, vivid descriptions of parents’ experiences using verbatim quotes helped to facilitate the transferability of data [51,52]. Copies of all transcripts, authors’ reflections, and any supporting documents were kept to maintain an audit trail and ensure the confirmability and dependability of the study [51,52]. All authors kept their critical reflections in individual web-based journals throughout the analysis process to enhance and ensure the reflexivity of this study’s findings [53,54].

### Ethical Considerations

Ethics approval for the main RCT with this nested study was obtained from the Domain Specific Review Board (reference number 2021/00227). Voluntary participation among participants was reinforced, and written informed consent was obtained before the study recruitment. Interviews were coded to maintain participants’ confidentiality. This unique code number was attached to the respective participant’s transcript. All consent forms were kept in a locked cupboard in the principal investigator’s office, while all audio recordings, survey responses, and transcripts were stored in a password-protected stand-alone computer.

## Results

### Participants’ Characteristics

Married fathers (10/20, 50%) and mothers (10/20, 50%) took part in this study. All parents except 1 (19/20, 95%) were employed. Parents from the control group had a mean age of 33.6 (SD 3.03) years, while parents in the intervention group had a mean age of 34.1 (SD 5.99) years. Of the 20 participants, most were Chinese (n=9, 45%), followed by Indian (n=5, 25%), Malay (n=4, 20%), Vietnamese (n=1, 5%), and White (n=1, 5%). In both groups, half of the parents (n=10, 50%) were first-time parents, while the rest (n=10, 50%) were experienced parents. Their mean parenting self-efficacy baseline scores ranged from 1.1 to 3.5 (total score range 1-4). More details of the parents’ characteristics and their PES scores are presented in Tables 1 and 2, respectively. The dropout rates of the main RCT were 16.9% (20/118) and 6.8% (8/118) for the intervention and control groups, respectively, at 1 month post partum and 8.5% (10/118) and 13.6% (16/118) for the intervention and control groups, respectively, at 3 months post partum.

**Figure 1:**
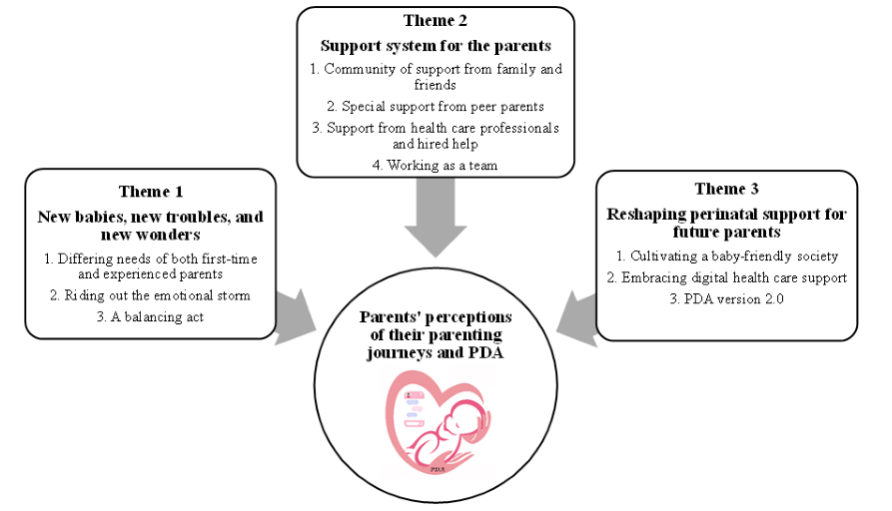
Summary of themes and subthemes. PDA: Parentbot–a Digital Healthcare Assistant.

Parents’ perceptions of the perinatal care received and their experiences with the PDA were captured in 3 main themes and 10 subthemes (Figure 1). The main themes were (1) new babies, new troubles, and new wonders; (2) support system for the parents; and (3) reshaping perinatal support for future parents.

**Table 1:** Participant characteristics (n=20).

Baseline characteristics			Intervention group (n=10)	Control group (n=10)			
Age (y), mean (SD)			34.1 (5.99)	33.6 (3.03)			
**Ethnicity, n (%)**							
	Chinese	5 (50)			4 (40)		
	Indian	3 (30)			2 (20)		
	Malay	1 (10)			3 (30)		
	Vietnamese	0 (0)			1 (10)		
	White	1 (10)			0 (0)		
**Marital status, n (%)**							
	Married	10 (100)			10 (100)		
Length of marriage (y), mean (SD)			6.0 (3.97)	4.9 (1.91)			
**Education level, n (%)**							
	Secondary school	1 (10)			1 (10)		
	ITE^a^, polytechnic, or junior college	1 (10)			0 (0)		
	University degree	6 (30)			7 (70)		
	Postgraduate degree	2 (20)			2 (20)		
**Employment status, n (%)**							
	Employed	9 (90)			10 (100)		
	Unemployed	1 (10)			0 (0)		
**Monthly household income (S** **GD),** **n (%)**							
	<3000	1 (10)			1 (10)		
	3000-7000	5 (50)			0 (0)		
	>7000	4 (40)			9 (90)		
**Baby’s gender, n (%)**							
	Male	6 (60)			5 (50)		
	Female	4 (40)			5 (50)		
**Childbirth method, n (%)**							
	Normal vaginal delivery	5 (50)			7 (70)		
	LSCS^b^ (elective)	3 (30)			1 (10)		
	LSCS (emergency)	1 (10)			2 (20)		
	Assisted vaginal delivery using vacuum extraction	1 (10)			0 (0)		
Attended antenatal preparation courses, n (%)			3 (30)	5 (50)			
**Did the mother follow the confinement period? n (%)**							
	Yes	4 (40)			5 (50)		
	No	1 (10)			0 (0)		
**Method of feeding baby, n (%)**							
	Breastmilk only (refers to both direct breastfeeding and expressed breastmilk)	5 (50)			4 (40)		
	A mixture of breastmilk and formula feeds	5 (50)			6 (60)		
**Length of maternity or paternity leave, n (%)**							
	<1 month	4 (40)			5 (50)		
	1-4 months	4 (40)			5 (50)		
	Did not take leave	1 (10)			0 (0)		
	Not applicable (not currently working)	1 (10)			0 (0)		
**Total number of children (including new baby), n (%)**							
	1	5 (50)			5 (50)		
	≥2	5 (50)			5 (50)		

^a^ITE: Institute of Technical Education.

^b^LSCS: lower segment cesarean section.

**Table 2:** Participants’ parenting self-efficacy scores at baseline.

Study participant code		Total parenting self-efficacy score (range 10-40)	Mean parenting self-efficacy score (range 1-4)
**Control group**			
	Mother control 1	28	2.8
	Mother control 2	20	2
	Mother control 3	30	3
	Mother control 4	28	2.8
	Mother control 5	29	2.9
	Father control 1	32	3.2
	Father control 2	20	2
	Father control 3	23	2.3
	Father control 4	11	1.1
	Father control 5	35	3.5
**Intervention group**			
	Mother intervention 1	27	2.7
	Mother intervention 2	30	3
	Mother intervention 3	29	2.9
	Mother intervention 4	31	3.1
	Mother intervention 5	35	3.5
	Father intervention 1	35	3.5
	Father intervention 2	26	2.6
	Father intervention 3	31	3.1
	Father intervention 4	20	2
	Father intervention 5	27	2.7

### New Babies, New Troubles, and New Wonders

Parents’ experiences and challenges faced while caring for their new baby were categorized into 3 subthemes: differing needs of both first-time and experienced parents, riding out the emotional storm, and a balancing act.

#### Differing Needs of Both First-Time and Experienced Parents

First-time parents from both the control and intervention groups experienced a “steep learning curve” during the first few weeks post partum as they learned the skills needed to care for their newborn (eg, breastfeeding and bathing). The parents from the control group felt confused by the conflicting advice received from websites on the internet, family and friends, and health care providers. They also often used “trial-and-error” means to identify the best methods to care for their child. Parents from the intervention group used the information provided by the PDA to improve their baby care skills and resolve the conflicting advice they received from other sources:

You don’t know what she wants whenever she cries...really you just have to figure it out each time...just trial and error.

Mother control 2

[T]he information provided in the app [PDA]...maybe it gives me 80% confidence to take care of my baby.

Father intervention 2

Although some experienced parents needed to refresh their memory of baby care skills, these parents had more confidence in caring for their newborn. However, they had to learn how to care for a new baby with unique needs and manage sibling rivalry. Those in the intervention group used the PDA to help them meet their evolving learning needs:

[M]y [first] child didn’t experience any like colic symptoms, so for the second one...I think experience colic all the time so I have to search like what to do...

Mother control 4

It’s quite helpful even for second time parents because like we will forget how to let’s say, take care of the umbilical cord and there’s some on sibling rivalry all that, that we can read on which is helpful for us.

Mother intervention 3

#### Riding Out the Emotional Storm

Parents from both groups experienced many positive and negative emotions within the first few weeks of postpartum. Their moods were closely tied to their abilities to care for the newborn; they felt stressed, helpless, and frustrated when they were unable to meet their newborn’s needs and felt joyous after learning how to care for their newborn sufficiently:

Like when the baby won’t stop crying, sometimes I get frustrated.... [But when] the problem is solved then it makes you feel better, gives you a boost that hey that you are capable, you are doing things right...

Father control 4

Parents from both groups managed their emotions by engaging in personal leisure activities (eg, watching Netflix, cooking, or cycling); planning their daily schedule carefully; and seeking professional help when needed. Those from the intervention group used the PDA’s activities (journal exercises, mindfulness exercises, and making and sharing of positive posters) to engage in self-reflection and regulate their thoughts and emotions:

Because people always post something positive and...you read, you think about it, ah, like that’s what exactly what you’re feeling now, and you feel happy about it. Yeah. This is very positive. I like this [the poster board].

Father intervention 5

#### A Balancing Act

Parents from both groups highlighted that they had to balance their work demands with baby care responsibilities. They worried about baby care arrangements after mothers’ maternity leave ended and applying for leave if their babies were to fall sick. Many parents wanted to forge a strong bond with their babies and were concerned that returning to work could cause them to miss their babies’ developmental milestones:

Maybe I’ll miss out on her milestones and all that...maybe at the end when she grows older, then I’ll regret not spending more time with her.

Father control 2

All parents also balanced their personal life and baby care by taking turns or engaging their family members (eg, mothers-in-law or mothers) to care for their baby:

Sometimes when my mother-in-law is free, she will help to take care of the baby. Me and my husband will go out, maybe just have a meal together.

Mother control 3

In addition, experienced parents from both groups had to balance the time spent with each of their children so that no child felt “neglected.” Experienced fathers often adopted the role of being the “main caregiver” for their older children, while experienced mothers mainly cared for the newborn due to their breastfeeding duties:

I had to take care more of the other kid...because she’s the one breastfeeding the other one...

Father control 5

### Support System for the Parents

The varied sources of support that parents had were organized into 4 subthemes: community of support from family and friends, special support from peer parents, support from health care professionals and hired help, and working as a team.

#### Community of Support From Family and Friends

Parents from both groups received valuable support from their family members (eg, parents, parents-in-law, and siblings) in terms of teaching them baby care skills, helping mothers recover after labor, and helping to care for their newborns. However, some parents from both groups had limited family support as their family members were busy with other responsibilities or unfit to care for newborns due to poor health. Moreover, some parents from both groups felt stressed when their older family members had different “outdated” methods of caring for babies that clashed with their views:

My mommy in-law is stubborn with her ways and plus that she also tends to be very critical in our processes at home...

Father control 5

[T]hey’re [own parents] quite old already, so I don’t really want to stress them too much, put too much strain on them.

Father control 3

In addition, parents from both groups valued hearing about their friends’ first-hand parenting experiences as it helped them to prepare for their baby’s birth and care for their baby:

We have a few friends who also just given birth...so we get to hear from them, their first-hand experience, what the husband has to do, what the wife has to do, what is the good to have things to be prepared...

Father control 3

#### Special Support From Peer Parents

Many mothers, mostly from the control group (5/5, 100%) and a few from the intervention group (1/5, 20%), sought support and advice from other local parents using web-based forums and WhatsApp groups, while fathers did not adopt this practice. However, parents were aware that these peers could sometimes provide “funny” advice:

I felt more comforted because a lot of people will just say that yeah I had my baby like hiccupping almost around the clock and they’re totally fine and so many people have that same experience.

Mother control 5

The poster board and support forum in the PDA provided parents in the intervention group with a sense of community. The simple interactions of making and sharing digital posters containing positive messages allowed parents to feel “less alone.” Although the support forum was underused, parents felt that the forum was a helpful and useful feature to have, especially if there was more activity on it:

If more people use [the support forum]...I think this will be a good way to find out from other parents or get some feedback from other parents on certain issues.

Father intervention 4

#### Support From Health Care Professionals and Hired Help

Parents from both groups appreciated the support provided by health care professionals at the hospital and clinics. Health care professionals answered parents’ queries thoroughly, taught them how to care for their babies patiently, and provided them with emotional support during their labor and inpatient stay. Some parents (mostly from the control group, 5/10, 50% and a few from the intervention group, 2/10, 20%) wanted more in-depth explanations from the physicians at the polyclinics to allay their anxieties:

The nurses were very nice...it was my first baby, so they actually taught me how to breastfeed, how to latch...When my son was crying, then I get overwhelmed...the nurse actually came by to help...

Mother intervention 1

Whatever questions we asked, the doctor [from polyclinic] would just say oh its normal normal normal, so we felt that it wasn’t really assuring.

Mother control 2

Confinement nannies helped parents from both groups by cooking nutritious meals for mothers to facilitate their postchildbirth recovery, taking care of newborns, and teaching parents how to care for their new babies. After the nannies left, some parents were sufficiently confident to care for their babies while, others (mostly from the control group and a few from the intervention group) were lost and stressed:

For the first month I hired a confinement nanny...Help to take care of the baby, feed the baby, uh, clothe the baby...She will slowly teach us la gradually.

Mother intervention 2

Domestic helpers mainly helped parents from both groups with household chores and meal preparations. While some parents from both groups also trained their helpers to care for their babies, the cultural differences between Singapore and the helpers’ own country made it challenging for helpers to assist with infant care:

We’re worried because they are from a different country...their ways of taking care and developing a baby might be more basic, more rudimentary.

Father control 4

#### Working as a Team

To support mothers, fathers from both groups spent more time at home by leaving work on time and seeking additional leave days. Fathers from both groups also supported mothers by doing household chores, caring for older children, and purchasing household and baby care necessities (eg, nursing pillows). Conversely, mothers from both groups took on more responsibility for their newborns during maternity leave so that fathers could have sufficient rest and focus on their work. In addition, all parents provided emotional support to each other and reminded one another that they were “both getting to be better parents day by day”:

[A]t the end of the working day, I just try to drop all my stuff and just run away, you know? Don’t care if work is incomplete, will just rush home.

Father control 2

Parents from the intervention group mostly used the PDA independently on their respective mobile phones and discussed the content they read to improve baby care:

I have missed out maybe example with the breastfeeding part...then [husband] come and share to me eh I have read it in Parentbot [PDA] they have stated like this, so you can try in this position, you can do it in this way.

Mother intervention 4

### Reshaping Perinatal Support for Future Parents

The different suggestions to improve perinatal care for parents were sorted into 3 subthemes: cultivating a baby-friendly society, embracing digital health care support, and PDA version 2.0.

#### Cultivating a Baby-Friendly Society

Parents from both groups would like to receive more financial assistance in the form of subsidies for baby supplies (eg, diapers, milk bottles, and milk powder) and infant care fees. To help parents balance their work demands and baby care, flexible work arrangements, longer and equal maternity and paternity leave (eg, 6 months), and more infant care slots were highlighted:

I think like a six month phase [leave] may even encourage more people to breastfeed at least up to that six month mark.

Mother control 5

That also calls for some changes where mother and father both are given equal leave. Then, the whole family gets involved in baby care because we get to leave, we are more in touch with the newborn.

Mother intervention 5

Parents from both groups also wanted to receive continuity of care during the perinatal period by having the same clinician during pregnancy, the same maternity team in the postnatal ward, and the same clinician caring for their baby at affordable subsidized rates. They also wanted babysitting and confinement nanny services to be subsidized and well regulated. Moreover, parents from both groups wanted to receive more holistic family-centered care by having their informational and emotional needs met and having their opinions respected by health care professionals:

[A]fter giving birth, if they could attach us to a family doctor...from the beginning, they will understand the history of the children more.... If we have continuity of care from the same doctor, it will be better.

Mother control 4

I had to insist to bring in the midwife earlier to check how dilated I am.... I had to ask three times before the nurse brought in the midwife.

Mother control 3

#### Embracing Digital Health Care Support

All parents in this study found it more convenient to search online for perinatal care information or use the PDA, as they could do their learning while commuting. Parents from the control group had difficulties searching for online validated information that applies to Singapore’s context, whereas those from the intervention group appreciated the contextualized trustworthy information provided by the PDA:

I found myself using this kind of on the go, like when I was sitting on the MRT [public train system in Singapore], I just scroll through and look for things.

Father intervention 3

Sometimes when I try to find things, it will show other country stuff.... I try to find just related to Singapore stuff and it’s scattered everywhere.

Mother control 1

Parents from the control group were interested in using telemedicine services and were open to the idea of communicating with chatbots as long as the computer-generated answers were prevetted by health care professionals. Parents from the intervention group who tried the chatbot in the PDA also had a pleasant experience interacting with it:

I think it’s quite interactive...there are one or two times I actually type in something, then the bot will actually reply to the questions.... I believe this one is already done with a lot of machine learning.... So answers are actually by the professional.

Father intervention 1

All the parents were interested in the PDA; when informed about the intervention, those from the control group wanted to try it, and all the parents from the intervention group would recommend the PDA to others. Parents from the intervention group indicated that the PDA was helpful for both fathers and mothers and both first-time and experienced parents. Those who received the PDA also wanted to have access to the PDA for a longer duration (eg, 6 months post partum) and wanted the PDA to provide more information about raising children throughout infancy and toddlerhood:

I think everybody should have it [PDA]...many of my friends who are becoming first-time mothers keep asking me, oh, what happens to this?... then I impart the same information, which I get from here [PDA]. [Mother intervention 5

#### PDA Version 2.0

Parents from the intervention group provided various suggestions to improve the PDA. They suggested using more “eye-catching” elements (eg, animations and brighter colors) to make the application more stimulating. Better organization and a more intuitive interface could help parents locate and use the PDA’s features more easily. Parents also found it “fun” to complete their assigned weekly activities in the PDA and see the corresponding colors of a digital rainbow badge light up (eg, green band will be filled in after completing a gratitude journal entry). However, they suggested having flexibility in the weekly activities and allowing users to customize their activities according to personal preferences to sustain their motivation to use the PDA:

So you can still have a rainbow badge, but maybe the violet is for if you complete one activity in the app, maybe the blue is...you can attach points to specific actions and then have them all add up until you get the full rainbow [badge].

Father intervention 3

To improve parents’ ease of access to information, the chatbot and search function in the PDA could be improved to locate more accurate answers to parents’ queries. The educational videos could be categorized into different sections for parents to easily access their segments of interest. Parents also requested to have more information on their emotional and mental health and possible pregnancy and infant complications:

For general questions, the chatbot is okay, but for more specific questions [e.g. How much weight should my baby gain in the first month?] it doesn’t answer...it keeps giving you the same answer.

Mother intervention 5

Parents also wanted to receive immediate replies (within 15 minutes) to their queries from health care experts (the standard reply was within 24 hours), feedback on their journal entries, and more encouragement on the poster board:

If possible, maybe you can have a live agent or somebody that we can talk to during working hours.

Mother intervention 3

Other features that parents would like to have in the PDA include baby care skills simulation games (eg, diaper changing and baby bathing) possibly using virtual reality technology, games to help parents with emotional regulation, and short quizzes on perinatal care knowledge with answers provided. Parents also wanted the PDA to track their pregnancy and children’s growth milestones and provide them with appropriate care tips at the respective time points:

[M]aybe the baby is crying and you have to figure out why it’s upset...So to have a game like that, it would help to put a lot of peoples’ fears at peace I think...

Father intervention 3

## Discussion

### Principal Findings

This study conducted a process evaluation of the PDA trial to explore the perinatal experiences and the user experiences of the PDA offered to parents in Singapore. Parents from different ethnic groups were interviewed, with Chinese, Malay, and Indian parents constituting most of the sample, which corresponds to the ethnic profile of Singapore [55]. There was also an equal mix of first-time and experienced parents from both the control and intervention groups who had a varied range of parenting self-efficacy scores at baseline.

All parents experienced positive and negative emotions during the first few weeks post partum, which is similar to previous research on local parents [19]. Although parents from both groups found ways to manage their negative emotions, parents in the intervention group had more options such as engaging in mindfulness-based and positive psychology (gratitude journal and poster making) activities in the PDA. Since these activities helped some parents to improve their psychological well-being as reported in previous research conducted in the United States and Europe [56,57], future perinatal interventions could include psychotherapeutic components to address parents’ psychological well-being [28]. However, as the PDA activities did not appeal to some parents, alternative activities such as games on emotional regulation were suggested by parents and could be explored by future studies, given that games could improve mental health outcomes among adults [58,59].

All parents in this study used web-based methods of learning perinatal care information (eg, websites, forums, and message groups) due to its convenience and accessibility. This ties in with the current generation of Singaporeans being more educated and encouraged to use digital technology in their daily lives [60,61]. Given that cultural norms affect perinatal care practices [62], parents benefited from receiving information that was relevant in the local context, a sentiment echoed by a previous local study [19]. Suggestions to improve parents’ PDA learning experience include quizzes, baby care simulation games (using virtual reality or digital animations), and personalized care tips being provided by push notifications at significant milestones during the perinatal period. Considering the prior success in using games to teach baby care skills (eg, breastfeeding) [63], future studies could develop more games to deliver perinatal education, especially in countries with good technological infrastructure such as Singapore [64]. Moreover, the tailoring of mobile app–based interventions’ content using individualized push notifications could improve participants’ engagement and the effectiveness of the intervention [65].

Parents from both groups expressed interest in communicating with chatbots, which ties in with the global trend of young adults being technology savvy and satisfied with interacting with artificial intelligence (AI) for their health needs [66,67]. Therefore, chatbots could supplement the current perinatal care by providing more in-depth information about parents’ health conditions to meet their needs [36], thereby addressing the manpower shortage faced by health care providers in Singapore and worldwide [68,69]. However, parents from the intervention group pointed out that the chatbot in the PDA could not sufficiently address all their queries, due to its lack of AI. This was a common problem reported by parents in a recent review, and chatbots were recommended to be programmed with natural language processing and AI to improve their communication skills [36]. Moreover, as parents in this study and previous research valued chatbots that were trustworthy and applicable to one’s culture [36], future chatbots could be preapproved by local health care professionals.

Parents from both groups received childcare support from older family members, especially their mothers and mothers-in-law, which is a common practice in Singapore and Asia [19,70]. While some parents valued the support provided by their family, others experienced distress due to their family’s differing views on childcare and hence preferred to seek advice from their friends who have had children recently and could provide up-to-date information. A similar finding was reported in a previous local study on parents [19]. Moreover, grandparents could also experience caregiver strain, especially if they have poor health conditions [71,72]. Some parents in this study were aware of this problem and decided to enroll their babies in infant care centers when they returned to work. Considering the current trend of couples delaying childbirth in Singapore [73], more grandparents might be older and have poorer health. Hence, more affordable and conveniently located infant care centers might be needed to meet the childcare needs of future local parents.

In addition to speaking with their friends, parents from both groups (especially mothers) sought peer support from web-based sources (eg, forums and messaging groups) to improve their socioemotional well-being. Those from the intervention group also benefited from their additional access to the PDA. Similar benefits of interacting with other parents have been noted in previous local studies [19,74]. According to the concept analysis of peer support by Dennis [75], peer support could effectively improve parents’ perinatal well-being by encouraging one’s help-seeking behaviors, allowing one to learn realistic coping methods, and inspiring one to adopt constructive and proper coping strategies undertaken by peers [76]. In the PDA, parents interacted with one another via a discussion forum and shared digital posters. While the poster board was generally enjoyed by the parents, the discussion forum was underused possibly due to the lack of moderation on the platform by researchers. To increase the forum’s activity, weekly topics could be introduced, and notifications or regular messages or posts could be initiated by researchers to encourage participants’ engagement [77].

While parents from both groups were generally satisfied with the current health care services, they would like to receive more continuity of care and family-centered care, which could lead to improved maternal and neonatal health outcomes [78,79]. In Singapore’s current public health system, parents do not receive care from the same team of health care professionals throughout the perinatal period. Hence, the provision of continuity of care could be enhanced by keeping detailed shared electronic records of mothers and babies and using collaborative care models [80]. Additional training of maternity health care professionals could be conducted to deliver respectful and holistic family-centered care [79]. Moreover, the implementation of midwifery-led care models to provide continuous high-quality maternal care throughout the perinatal period could be explored and promoted in Singapore and on a global scale [81-84].

Current findings highlighted the importance of parents’ support for each other. The PDA promoted partner support by encouraging parents to discuss the information they read and learn tips on supporting one another, which was similar to the findings from previous local studies [19,33]. As receiving support from one’s partner can improve one’s psychological well-being and parenting behaviors [85,86], future perinatal interventions delivered to both parents should continue to focus on this aspect to optimize their benefits. Moreover, many fathers in this study showed interest in being involved with childcare. The shift in fathers’ mindset from the traditional breadwinner role to taking on more active roles in childcare was also reported in other parts of Asia (eg, Thailand, Japan, and Taiwan) [87,88] possibly due to couples adopting more egalitarian relationships [89].

With fathers wanting to be more involved in childcare duties, mothers and fathers from both groups in this study were concerned about balancing their work demands and baby care responsibilities. This is a common worry among parents with young children [90], and employers in Singapore have been looking into establishing flexible work arrangements especially after the COVID-19 pandemic to help parents achieve a healthy work-family balance [91,92]. Parents also requested longer maternity and paternity leaves for up to 6 months to encourage exclusive breastfeeding for the first 6 months of an infant’s life [93], especially since only 40% of mothers in Singapore exclusively breastfeed their children for the first 6 months post partum [94]. An equal duration of paternity and maternity leaves was also suggested, which align with the current generation of parents wanting to assume more equal responsibility for their children’s well-being [89]. As paternal involvement significantly contributes to better maternal and child health outcomes [95], Singapore has recently doubled paternity leave from 2 to 4 weeks to encourage more father-infant bonding [96]. Plans to further lengthen paternity and maternity leaves and its influence on parenting and newborn outcomes could be explored.

### Strengths and Limitations

This study provided valuable insight into the parenting experiences of first-time and experienced parents with perinatal care in the current digital age and their user experiences of the newly developed PDA intervention. It explored how mobile app–based support and chatbots could enhance perinatal care. Current findings are important in guiding future research on the provision of technology-based perinatal support for parents in Asia, especially using mobile apps and chatbots.

However, this study has some limitations. As only married English-speaking heterosexual parents who had a newborn without serious medical complications were recruited from a single tertiary hospital, the findings of this study may not represent the views of all parents in Singapore. Although data saturation was achieved, the relatively small sample size for this study could have hindered the transferability of its findings to all parents in Singapore. Since this study was conducted on multiracial parents in Singapore, its results might not be transferable to parents in other contexts. Moreover, with all study participants identifying as heterosexual, the findings of this study might not be representative of parents from the lesbian, gay, bisexual, transgender, and queer (LGBTQ) community. While some gender-specific and parity-specific differences in the perinatal experiences of parents were reported in this study, more in-depth gender and parity differences could not be explored due to the nature of this study with small sample size. In addition, the use of only personal interviews in this study could introduce self-reported bias and impair the confirmability of the findings. As this study only recruited a convenient sample of parents who had smartphones and were willing to participate, other parents who did not own smartphones (rarely seen in the Singapore context) or who had actual needs but were not keen to participate in research studies could be underrepresented in this study. Furthermore, since all study participants were only interviewed once between 1 and 3 months after childbirth, this study’s findings do not reflect parents’ experiences across the entire perinatal period. Finally, due to logistical limitations (limited grant funding and resources), the research team could not feasibly develop the PDA to provide support for parents until 1 year post partum, and hence this study could not explore parents’ needs and experiences throughout the perinatal period.

### Implications for Future Research and Practice

Considering the unmet needs of new parents and that the PDA can provide all parents with informational, socioemotional, and psychological benefits, mobile app–based perinatal interventions with in-built chatbots could be used to supplement the perinatal care provided for parents in Singapore. To optimize performance, future chatbots could have natural language processing and AI capabilities and have their answers vetted by local health care professionals. Future interventions could include more peer-to-peer interactions, games on emotional regulation and baby care skills, quizzes on perinatal care information, and personalized information for parents at significant milestones. Health care professionals could adopt midwife-led continuity of care models to deliver family-centered care. To address the parents’ childcare needs, longer and equal paternity and maternity leaves could be explored, and flexible work arrangements could be promoted. Future research on more diverse parents (eg, from the LGBTQ community; single parents; unmarried parents; parents who had newborns with medical complications; and non–English-speaking parents) could be conducted to obtain a more holistic understanding of local parents’ perinatal experiences. Educational resources in the PDA would also have to undergo revisions to meet the needs of varied family compositions and parents whose babies have medical complications. For instance, advice on bonding with a nonbiological child and tips on caring for children with medical complications could be provided. Future longitudinal mixed methods studies on the perinatal experiences of parents could also be conducted to triangulate the findings and obtain more in-depth data. These mixed methods studies could consider conducting quantitative surveys among larger sample of parents from both control and intervention groups to further compare their experiences and satisfaction in using the PDA. Future research could recruit larger sample sizes, explore gender- and parity-specific differences more in depth, and use more methods to collect qualitative data from participants such as observations. Furthermore, future trials could aim to develop and evaluate similar technology-based interventions for parents up to 1 year post partum to obtain a more holistic understanding of their experiences throughout the perinatal period. Finally, future research could engage grandparents to explore their views regarding childcare since they are also involved in caring for children in Singapore.

### Conclusions

Overall, this study showed that the PDA can provide parents with informational, socioemotional, and psychological support during the perinatal period. Despite its limitations, this study has paved the way for the development of better mobile app–based interventions and chatbots to meet the needs of Asian parents during the perinatal period. Moving forward, researchers would need to overcome the problems posed by the limited available resources (funds, technological capabilities, and manpower) to design future perinatal supportive interventions with a more sophisticated chatbot, more gamification features, personalized and engaging care tips, and more peer-to-peer interactions. Health care providers could focus on delivering more family-centered, holistic, and continuous care for parents and babies. Policy makers and employers could be encouraged to help parents achieve a healthy work-family balance. Finally, collaboration between health care providers, researchers, and technical experts would be needed to develop appropriate and effective chatbot- and mobile app–based interventions to supplement perinatal care shortly.

## Abbreviations

AI: artificial intelligence
COREQ: Consolidated Criteria for Reporting Qualitative Research
LGBTQ: lesbian, gay, bisexual, transgender, and queer
PDA: Parentbot–a Digital Healthcare Assistant
PES: Parenting Efficacy Scale
RCT: randomized controlled trial
